# Efficacy and Safety of Cannabinoids for Autism Spectrum Disorder: An Updated Systematic Review

**DOI:** 10.7759/cureus.80725

**Published:** 2025-03-17

**Authors:** Danilo A Pereira, Lidia Cheidde, Mateus Daniel R Megiolaro, Ana Elisa F Camargo, Elizabet Taylor P Weba, Victor G Soares, Anderson M Pereira da Silva, Laura Cheidde, Pedro Paulo Ladeira Júnior, Dillan C Amaral, Rafael Triaca, Matheus Augusto N Fernandes, Paula Maria P Mimura

**Affiliations:** 1 Department of Human Reproduction and Childhood, Pontifícia Universidade Católica de São Paulo, Sorocaba, BRA; 2 Faculty of Medicine, Pontifícia Universidade Católica de São Paulo, Sorocaba, BRA; 3 Faculty of Medicine, Universidade Estadual da Região Tocantina do Maranhão, Imperatriz, BRA; 4 Faculty of Medicine, Universidade Federal dos Vales do Jequitinhonha e Mucuri, Diamantina, BRA; 5 Department of Pharmacology, Universidade Federal do Vale do São Francisco, Petrolina, BRA; 6 Department of Orthopaedics and Trauma, Universidade Cidade de São Paulo (UNICID), São Paulo, BRA; 7 Faculty of Medicine, Universidade Cidade de São Paulo (UNICID), São Paulo, BRA; 8 Faculty of Medicine, Universidade Federal do Rio de Janeiro, Rio de Janeiro, BRA; 9 Faculty of Medicine, Universidade Unigranrio Escola de Ciências da Saúde, Duque de Caxias, BRA; 10 Faculty of Medicine, Universidade Anhembi Morumbi, São José dos Campos, BRA; 11 Department of Specialized Rehabilitation, Hospital Estadual Especializado em Reabilitação "Dr. Francisco Ribeiro Arantes", Itu, BRA

**Keywords:** a systematic review, autism spectrum disorder, cannabinoids, efficacy, safety

## Abstract

Autism Spectrum Disorder (ASD) lacks an established pharmacological treatment protocol, prompting interest in alternative therapeutic approaches, such as cannabidiol (CBD). This systematic review evaluates the potential efficacy and safety of CBD-rich formulations in managing ASD symptoms. A comprehensive search of PubMed, Embase, Scopus, Web of Science, and the Cochrane Library identified seven studies encompassing 494 patients from Brazil and Israel. Preliminary findings suggest that CBD-rich formulations may provide modest benefits for sleep and social interaction, with a reduction in anxiety symptoms. Regarding core ASD symptoms and behavioral outcomes, cannabinoids demonstrated greater efficacy compared to placebo in some studies. However, adverse events varied, and response to treatment was inconsistent across individuals. While cannabinoids, particularly CBD-rich formulations, appear to be relatively safe and potentially beneficial, further large-scale, controlled trials comparing CBD to established ASD treatments are essential to clarify its role and long-term impact in ASD management.

## Introduction and background

Autism Spectrum Disorder (ASD) is a complex neurodevelopmental condition characterized by difficulties in social communication and restricted, repetitive behaviors [1]. It affects about 1%-2% of children worldwide, with recent estimates in the United States indicating that approximately 1 in 36 children is diagnosed with ASD [2]. Despite the growing prevalence of ASD, there are currently no established pharmacological treatments for its core symptoms [3]. Clinicians often manage associated behaviors (such as irritability, hyperactivity, or anxiety) with medications like antipsychotics or antidepressants, but these provide limited benefit and can cause significant side effects (e.g., weight gain and sedation) [3]. This therapeutic gap has led families and clinicians to seek alternative interventions, including cannabinoid-based therapies.

Cannabinoids have emerged as a potential therapeutic option due to their interaction with the endocannabinoid system (ECS), which regulates brain function, mood, and immune responses. The primary receptors, CB1 and CB2, are involved in neurotransmitter modulation and neuroinflammation, both of which are implicated in ASD [4,5]. Studies have reported lower endocannabinoid levels in children with ASD, suggesting that ECS dysfunction may contribute to symptoms [6,7]. Additionally, cannabidiol (CBD) interacts with serotonin (5-HT1A) and PPAR receptors, which may underlie its potential anxiolytic and neuroprotective effects [7-9].

Previous research on cannabinoids in ASD highlights both a potential therapeutic signal and notable gaps in knowledge. Early studies suggest that cannabinoids (particularly CBD-enriched preparations) may help reduce irritability, aggression, anxiety, and other behavioral problems in some individuals with ASD and generally appear to be well tolerated. The mechanistic rationale - involving the ECS and related pathways - provides a compelling reason to explore this treatment, yet it also underscores the complexity of how cannabinoids might interact with neurodevelopmental processes.

Given the increasing use of cannabinoids by ASD patients and the limited high-quality evidence, this systematic review updates the literature, aiming to evaluate the efficacy and safety of cannabinoids in ASD [10,11].

## Review

Methods

Protocol

This systematic review was performed following the handbook of the Cochrane Collaboration [12] and the Preferred Reporting Items for Systematic Reviews and Meta-Analysis (PRISMA) statement guidelines [13].

Search Strategy and Data Source

We systematically searched PubMed, Embase, Web of Science, Scopus, and Cochrane databases. Our search was last updated on January 28th, 2025. The complete search strategy is: ("Autism Spectrum Disorder" OR "ASD") AND ("Cannabinoids" OR Cannabidiol OR "CBD" OR "THC" OR "Tetrahydrocannabinol"). All records retrieved were independently assessed by three authors (L.C., M.D.R.M., and D.C.A.). Full texts arbitrated a decision regarding full-text retrieval were reviewed by L.C. and M.D.R.M., and discussed regarding inclusion and exclusion criteria. 

The articles were uploaded to the Rayyan platform (Qatar Computing Research Institute, Ar-Rayyan, Qatar) for title and abstract reading. The first selection was focused on the title and abstract, with no limitations on the publication date. In this stage, all duplicated titles were removed, and articles that did not directly address the subject of interest were excluded. Two reviewers (L.C. and M.D.R.M.) performed this step independently; doubts were clarified with the aid of a third researcher (D.C.A.). Full texts were reviewed by L.C. and M.D.R.M. and discussed regarding inclusion and exclusion criteria. Full texts of potential articles were read in their entirety. Reference lists of eligible studies and relevant reviews were searched for additional articles.

Eligibility Criteria - Inclusion and Exclusion Criteria

We included randomized clinical trials (RCTs) and observational studies that investigated the use of cannabinoids in patients with ASD, with reports of at least one outcome of interest. Studies were excluded if they did not assess any relevant efficacy or safety outcomes, were not published in English, involved animal or in vivo models, or were classified as reviews, case reports, abstracts, protocols, letters, or comments.

Data Extraction and Outcomes Assessment

The following data were extracted from the selected articles: authors, year of publication, study location, type of study, sample size and age, patient characteristics, duration of intervention, therapeutic scheme, follow-up time, and main results. Two reviewers (E.T.P.W. and P.P.L.J.) were responsible for extracting and managing the data independently, which were inserted into an EXCEL® spreadsheet (Microsoft® Corp., Redmond, WA, USA). Doubts were clarified with the help of the third researcher (L.C.).

The included studies assessed various clinical outcomes related to the efficacy and safety of cannabinoids in patients with ASD. The primary outcomes analyzed were sleep quality, core autism symptoms, behavior, social effects, anxiety, and adverse events. No specific measurement scales were excluded, ensuring a comprehensive assessment of these clinical parameters.

Risk of Bias Assessment

We evaluated the risk of bias using version 2 of the Cochrane Risk of Bias assessment tool [12] and the updated version of the Cochrane Risk of Bias in Non-randomized Studies of Interventions tool (ROBINS-I) [13]. Two authors independently assessed the studies for quality assessment (E.T.P.W. and V.G.S.), and any conflict was resolved by a third author (L.C.).

Results

Study Selection and Characteristics

**Figure 1:**
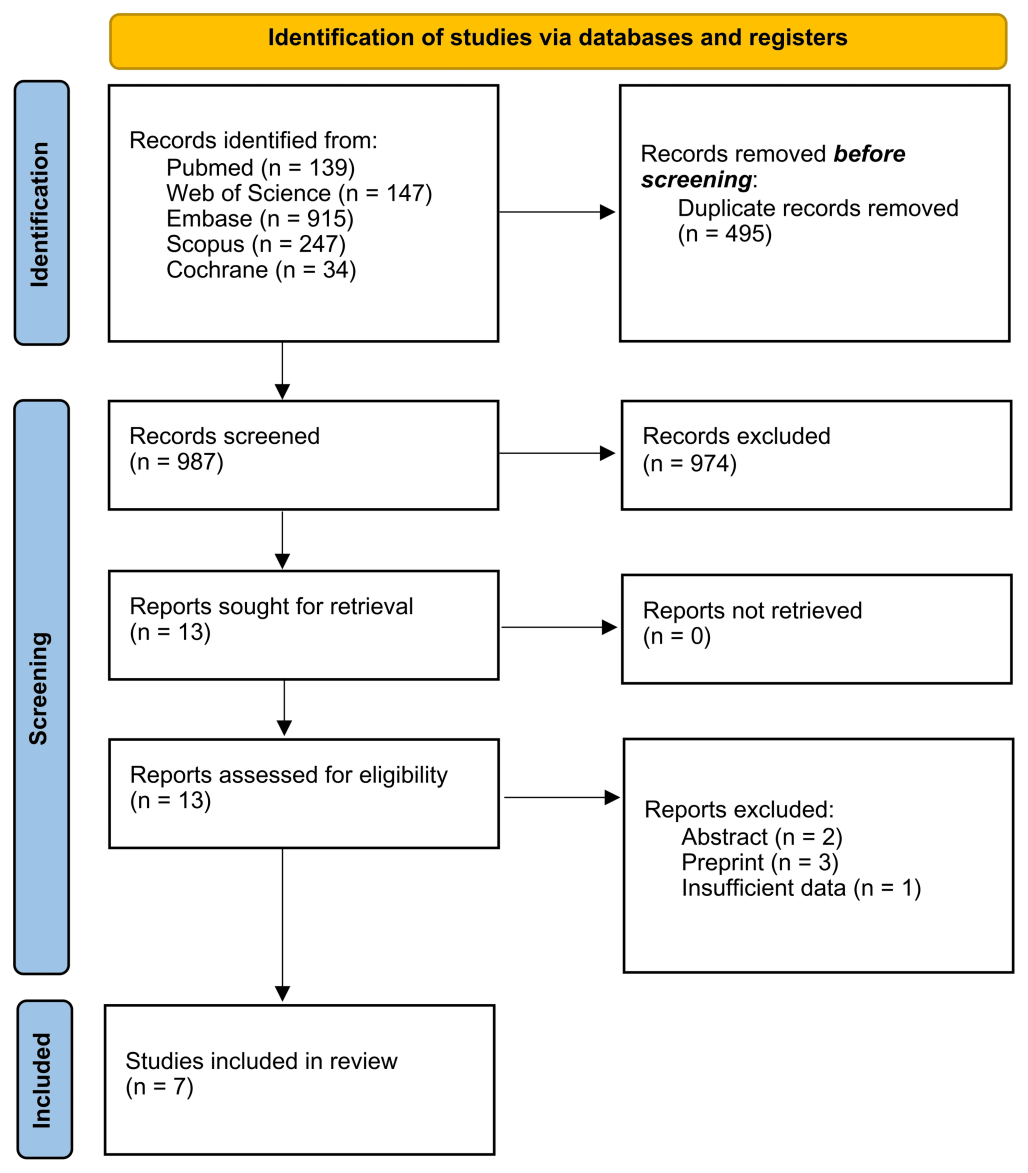
PRISMA Flow Diagram PRISMA: Preferred Reporting Items for Systematic Reviews and Meta-Analysis

A total of 1,509 studies were identified: 139 from PubMed, 147 from Web of Science, 915 from Embase, 247 from Scopus, and 34 from Cochrane. After the removal of duplicates, 987 studies remained for the title and abstract screening. Subsequently, 13 articles underwent a full-text review, and six were excluded due to incompatibility with the eligibility criteria. Therefore, seven studies were included [3,14-19]. The study selection process is illustrated in Figure 1.

Seven studies were included in this review, involving 494 patients from Israel and Brazil. The mean age of participants ranged from 7.68 to 12.9 years. The proportion of male participants was high (n = 366, or 74.01%). Autism severity was mostly classified as severe or was not reported. Concomitant medications included antipsychotics, mood stabilizers, stimulants, and benzodiazepines. It should be noted that the Aran et al. [3] and Schnapp et al. [15] studies are part of the same clinical trial and, therefore, have the same intervention protocols for the same patients (Table 1).

**Table 1:** Baseline Characteristics Note: Aran et al. [3] and Schnapp et al. [15] analyze the same population and intervention but assess different outcomes, which justifies including both. However, their patients were counted only once. CGI-S: Clinical Global Impression Scale-Severity; SSRI: Selective Serotonin Reuptake Inhibitor; NR: Not Reported

Author (Year)		Country	Study Design	Total Patients	Mean Age ± SD	Male/Female	Autism Severity	Concomitant Medications (No. of Patients)
Junior et al. (2024) [14]		Brazil	Randomized Clinical Trial	60	7.68 ± 1.74	52/8	Mild (n = 26), Moderate (n = 29), Severe (n = 5)	Non-specified (n = 28)
Aran et al. (2019) [19]		Israel	Retrospective Cohort Study	60	11.8 ± 3.5	50/10	Severe (CGI-S: 6/7)	Antipsychotics (n = 43), Mood stabilizers (n = 10), Benzodiazepines (n = 7), SSRI (n = 4), Stimulants (n = 4)
Aran et al. (2021) [3]		Israel	Randomized Clinical Trial	150	11.8 ± 4.1	120/30	Severe	Antipsychotics (n = 18), Anticonvulsants (n = 10), Benzodiazepines (n = 5), SSRI (n = 21), Stimulants (n = 20), Melatonin (n = 12)
Schnapp et al. (2022) [15]		Israel	Randomized Clinical Trial	150	11.8 ± 4.1	120/30	Severe	Antipsychotics (n = 18), Anticonvulsants (n = 10), Benzodiazepines (n = 5), SSRI (n = 21), Stimulants (n = 20), Melatonin (n = 12)
Barchel et al. (2019) [18]		Israel	Prospective Cohort Study	54	11.3 ± 3.96	45/8	NR	Stimulants (n = 5), Antipsychotics (n = 37), Anti-epileptics (n = 8), Melatonin (n = 4), Antidepressants (n = 2), Other anti-muscarinics (n = 3), Alpha agonist (n = 1)
Hacohen et al. (2022) [16]		Israel	Prospective Cohort Study	82	9.3 ± 0.5	44/38	NR	NR
Bar-Lev Schleider et al. (2019) [17]		Israel	Retrospective Cohort Study	188	12.9 ± 7.0	154/34	NR	Antipsychotics (n = 55), Anti-epileptics (n = 46), Antidepressants (n = 10), Hypnotics and sedatives (n = 10), Anxiolytics (n=7)

*Intervention Description* 

Table 2 summarizes the intervention types, dosing schedules, and concentration details, highlighting the differences and methodologies employed across the studies.

**Table 2:** Intervention and Posology Description **Aran et al. [3] and Schnapp et al. [15] analyze the same population and intervention, but assess different outcomes, which justifies including both. However, their patients were counted only once; ***No other details were provided. CBD: Cannabidiol; THC: Tetrahydrocannabinol; CBD:THC: Cannabidiol to Tetrahydrocannabinol Ratio

Author (Year)	Intervention Description	Posology	Route Administration	Follow-Up
Junior et al. (2024) [14]	31 CBD-rich cannabis extract (ratio of 9CBD:1THC)/29 Control (placebo) from ABRACE	Starting dose: 3 drops every 12 hours, titrated by 2 drops every 12 hours twice a week, up to max 70 drops/day	Orally	12 weeks
Aran et al. (2021) [3]	50 whole-plant cannabis extract (ratio of 20CDB:1THC) (BOL-DP-O-01-W, BOL Pharma, Israel)/50 Purified CBD/THC extract (ratio of 20CDB:1THC) (BOL-DP-O-01-W, BOL Pharma, Israel)/50 Control (placebo)**	Starting dose: 1 mg/kg/day CBD + 0.05 mg/kg/day THC, increased by 1 mg/kg/day CBD + 0.05 mg/kg/day THC every other day. Maximum dose: 10 mg/kg/day CBD + 0.5 mg/kg/day THC (max 420 mg CBD, 21 mg THC/day) for children 20-40 kg; 7.5 mg/kg/day CBD + 0.375 mg/kg/day THC for children >40 kg.	Orally (sublingual whenever possible)	12 weeks
Schnapp et al. (2022) [15]	50 whole-plant cannabis extract (ratio of 20CDB:1THC) (BOL-DP-O-01-W, BOL Pharma, Israel)/50 Purified CBD/THC extract (ratio of 20CDB:1THC) (BOL-DP-O-01-W, BOL Pharma, Israel)/50 Control (placebo)**	Starting dose: 1 mg/kg/day CBD + 0.05 mg/kg/day THC, increased by 1 mg/kg/day CBD + 0.05 mg/kg/day THC every other day. Maximum dose: 10 mg/kg/day CBD + 0.5 mg/kg/day THC (max 420 mg CBD, 21 mg THC/day) for children 20-40 kg; 7.5 mg/kg/day CBD + 0.375 mg/kg/day THC for children >40 kg.	Orally (sublingual whenever possible)	12 weeks
Aran et al. (2019) [19]	60 whole-plant cannabis extract (ratio of 20CBD:1THC) from multiple suppliers (CHP, Tikun Olam, BOL Pharma)	Starting dose: 1 mg/kg/day of CBD, two to three times per day. Maximum dose: 10 mg/kg/day of CBD two to three times per day.***	Sublingually	10.9 ± 2.3 months
Bar-Lev Schleider et al. (2019) [17]	188 cannabis oil solution (30% CDB and 1.5% THC, ratio of 20CBD:1THC)	Starting dose: 1 drop (0.05 mL) three times a day (each drop contains 15 mg CBD and 0.75 mg THC). Titration was individualized based on patient response. Max dose: up to 20 drops three times a day.	Sublingually	6 months
Hacohen et al. (2022) [16]	110 whole-plant cannabis extract (ratio of 20CDB:1THC) (Nitzan Spectrum®, Search Medical Group, Israel)	Starting dose: 1 drop daily (each drop contains 0.3 mg THC and 5.7 mg CBD), gradually increased until improvements were noted. Maximum dose: 10 mg/kg/day CBD (max 400 mg/day) and 0.5 mg/kg/day THC (max 20 mg/day).	Orally	6 months
Barchel et al. (2019) [18]	53 cannabinoid oil solution (30% CBD, 20:1 CBD:THC ratio) from Tikun Olam	Starting dose: Individualized titration, recommended daily dose to 16 mg/kg/day CBD (max 600 mg/day) and 0.8 mg/kg/day THC (max 40 mg/day)	Oral (sublingual drops)	10.15 ± 5.29 months

Anxiety

RCT studies: In the Junior et al. [14] study, anxiety levels were assessed using a non-specific semi-structured questionnaire. The CBD-rich cannabis group showed a significant reduction in anxiety, with a mean score of 1.84 (±1.39) compared to 2.90 (±1.23) in the placebo group (p = 0.0159).

In contrast, Aran et al. [3] and Schnapp et al. [15] are part of the same RCT but analyzed different outcomes. Schnapp et al. [15] focused on sleep-related anxiety using the Children’s Sleep Habit Questionnaire (CSHQ) and found no significant difference (p = 0.59) in anxiety scores between the CBD and placebo groups (CBD = -0.4 ± 1.2; placebo = -0.6 ± 1.3). Additionally, Aran et al. [3] reported anxiety-related adverse events across treatment groups. In the whole-plant extract group, 20% (10 out of 50) of participants experienced adverse anxiety-related effects, while in the pure cannabinoids group, 27% (14 out of 50) were affected. In contrast, 14% (7 out of 50) of participants in the placebo group also reported anxiety-related adverse events.

Observational studies: Among observational studies, Barchel et al. [18] used a parent-reported symptom questionnaire and found that, among 17 participants, 47.1% (8 out of 17) showed improvement, while 29.4% (5 out of 17) had no change, and 23.5% (4 out of 17) experienced worsening. Similarly, Bar-Lev Schleider et al. [17] used a parent-reported treatment effectiveness scale and found that, among 27 patients receiving cannabinoid oil, 88.8% (24 out of 27) showed improvement, while 11.1% (3 out of 27) had no change or worsening. Hacohen et al. [16] used the Autism Diagnostic Observation Schedule (ADOS-2) and reported that anxiety was indirectly affected, with larger social communication improvements noted in those with more severe initial symptoms. However, this study did not directly measure anxiety as an independent outcome.

Sleep

RCT studies: In the Junior et al. [14] study, sleep quality was assessed using a non-specific semi-structured questionnaire. The CBD-rich cannabis group showed an improvement in sleep scores, with a mean change of 0.77 (±1.61) in the cannabis group, compared to 0.28 (±0.59) in the placebo group (p = 0.0711), though the result did not reach statistical significance. Meanwhile, Aran et al. [3] and Schnapp et al. [15] are from the same RCT, analyzing different aspects of sleep disturbances using the CSHQ. Schnapp et al. [15] found no significant improvement in sleep parameters with cannabinoids compared to placebo. For total sleep scores, the mean change was -2.9 (±9.2) in the pure cannabinoids group, -2.3 (±5.6) in the whole-plant extract group, and -1.4 (±6.6) in the placebo group (p = 0.63), indicating no statistical difference. 

Observational studies: In Barchel et al. [18], a parent-reported questionnaire revealed that, among 21 participants with reported sleep issues, 71.4% (15 out of 21) showed improvement, while 23.8% (5 out of 21) had no change, and 4.7% (1 out of 21) experienced worsening. Similarly, Bar-Lev Schleider et al. [17] used a parent-reported treatment effectiveness scale and found that, among 27 patients using cannabinoid oil, 88.8% (24 out of 27) showed improvement in sleep, while 11.1% (3 out of 27) had no change or worsening. Hacohen et al. [16] did not directly measure sleep outcomes but reported that parents noted general behavioral improvements, including sleep disturbances, among children with more severe baseline symptoms. However, without direct sleep assessments, it remains unclear if cannabinoids specifically influenced sleep or if improvements were secondary to other behavioral changes. The Aran et al. [19] study reported caregiver-perceived improvements in sleep as part of a broader clinical evaluation but did not provide specific quantitative sleep measures.

Autistic Core Symptoms

RCT studies: In the Junior et al. [14] study, autistic core symptoms were assessed using the Autism Treatment Evaluation Checklist (ATEC) and the Childhood Autism Rating Scale (CARS). The ATEC total score at the final assessment was 64.84 (±26.82) in the CBD-rich cannabis group and 75.00 (±32.89) in the placebo group, with the difference not being statistically significant (p = 0.098). Similarly, the CARS final score was 33.47 (±8.48) in the cannabis group and 37.83 (±9.02) in the placebo group, with the difference also not being statistically significant (p = 0.188). The Aran et al. [3] and Schnapp et al. [15] studies originate from the same RCT, analyzing autistic core symptoms using the Autism Parenting Stress Index (APSI). In the Aran et al. [3] trial, whole-plant cannabis treatment resulted in a change from a baseline of -6.73 (±11.58) in APSI scores, while the pure cannabinoid group had no reported baseline scores. The placebo group showed a smaller APSI change of -1.76 (±10.25), suggesting greater improvement in the whole-plant cannabis group; however, the p-value was not significant in comparison with whole-plant and pure cannabis versus placebo (p = 0.502 and p = 0.513).

Observational studies: Aran et al. [19] reported that whole-plant cannabis reduced APSI scores from 2.04 (±0.77) at baseline to 1.37 (±0.59) at follow-up, indicating an improvement of 0.66 ± 0.74 in autism-related parental stress. In Hacohen et al. [16], autistic symptoms were evaluated using ADOS-2. The ADOS-2 total score showed a mean change of -0.56 (±0.17) from baseline in the CBD-rich cannabis group, while baseline values and placebo group scores were not reported. The Social Responsiveness Scale (SRS-2) total score improved by -3.29 (±1.13), and the Vineland Adaptive Behavior Scale (Vineland-3) total score increased by +4.37 (±1.18), indicating mild improvements in adaptive behavior. In Barchel et al. [18], a parent-reported questionnaire found that 74.5% of children showed improvement in autistic symptoms, while 21.6% had no change, and 3.9% experienced worsening. Similarly, Bar-Lev Schleider et al. [17] used a parent-reported effectiveness scale and found that 91.4% (of an unspecified number of participants) reported improvements, while 8.6% saw no change.

Social Effects

RCT studies: In the Junior et al. [14] study, social interaction was measured using an unspecified scale. The CBD-rich cannabis group had a final score of 1.68 (±1.01), while the placebo group scored 2.83 (±1.10), indicating better social outcomes in the cannabis group (p < 0.05). The ATEC Socialization subscale showed similar trends, with the CBD-rich cannabis group scoring 13.64 (±6.31) compared to 17.83 (±9.83) in the placebo group; however, the p-value was 0.113.

The Aran et al. [3] and Schnapp et al. [15] studies originate from the same RCT, analyzing social effects using the SRS-2. Aran et al. [3] reported that the whole-extract cannabis group showed a mean change from baseline of -14.9 (±14.34), the pure cannabinoid group showed a mean change from baseline of -9.13 (±28.33), while the placebo group improved by -5.85 (±23.17) in SRS-2 scores, suggesting a significant benefit of whole-extract (p = 0.009) and a moderate but non-significant benefit of pure cannabinoids versus placebo (p = 0.801).

Observational studies: In Hacohen et al. [16], in the CBD-rich cannabis group, social symptoms were evaluated using the ADOS-2 Social Affect Score, which improved by -0.49 (±0.18) from baseline, and the SRS-2 Social Score, which improved by 2.51 (±1.19). These findings suggest a possible positive impact of cannabinoids on social responsiveness, though placebo data were not provided for direct comparison. In Barchel et al. [18], a parent-reported questionnaire showed that 71.4% of children displayed improvement in social interaction, while 23.8% showed no change, and 4.7% experienced worsening. Similarly, Bar-Lev Schleider et al. [17] used a parent-reported effectiveness scale but did not provide numerical data on social improvements​.

Behavioral Outcomes

RCT studies: In the Junior et al. [14] study, behavior was assessed using multiple measures. The ATEC Health and Behavior subscale showed a final score of 25.35 (±10.79) in the CBD-rich cannabis group and 27.17 (±11.03) in the placebo group (p = 0.119). Similarly, psychomotor agitation scores were lower in the cannabis group (1.64 ± 1.28) compared to placebo (2.65 ± 1.14) (p = 0.00295), and stereotypy scores were also reduced in the cannabis group (1.45 ± 1.06) versus placebo (2.07 ± 1.03) (p = 0.3853), suggesting a potential benefit of cannabinoids in reducing repetitive and hyperactive behaviors. However, only the psychomotor agitation was statistically significant. The Aran et al. [3] and Schnapp et al. [15] studies originate from the same RCT, analyzing behavioral effects using the Clinical Global Impression-Improvement (CGI-I) scale. Aran et al. [3] found that 49% of participants in the whole-plant cannabis group and 38% in the pure cannabinoid group were rated as “much improved” or “very much improved,” compared to 21% in the placebo group (p = 0.005). However, changes in Home Situation Questionnaire-ASD (HSQ-ASD) scores did not differ significantly between groups (p > 0.05), suggesting that caregiver-reported disruptive behaviors did not improve consistently​.

Observational studies: In Aran et al. [19], 61% of participants using whole-plant cannabis were rated as improved in behavior problems on the Caregiver Global Impression of Change (CGIC) scale. Similarly, Barchel et al. [18] reported that parents observed improvements in aggression and behavioral regulation, though specific numerical data were not provided. In Hacohen et al. [16], behavior was evaluated using the SRS-2 Restricted and Repetitive Behavior (RRB) score, which showed a mean improvement of -2.88 (±1.14) in the CBD-rich cannabis group. Finally, Bar-Lev Schleider et al. [17] did not provide specific quantitative data on behavioral outcomes.

Adverse Events

In the studies that included a comparison between the intervention and placebo, the number of adverse events was very similar between the control and intervention groups, indicating the safety of the intervention. In the Junior et al. [14] and Schnapp et al. [15] studies, the adverse effects were not specified individually; only the total number of events, over the total number of patients, was provided. Table 3 reports the summary of adverse events.

**Table 3:** Summary of Adverse Events **Drop-out participants Note: Aran et al. [3] and Schnapp et al. [15] adopt a two-phase crossover approach, which justifies the doubling of the total number of events in relation to the study population. NR: Not Reported; CBD: Cannabidiol

Study (Year)	CBD (Number of Events)	Control (Number of Events)	Treatment Duration
Aran et al. (2021) [3]	Somnolence: 51, Decreased appetite: 46, Weight loss: 25, Tiredness: 59, Euphoria: 39, Anxiety: 47	Somnolence: 7, Decreased appetite: 14, Weight loss: 4, Tiredness: 19, Euphoria: 13, Anxiety: 14	3 months
Junior et al. (2024) [14]	Dizziness, insomnia, colic, and weight gain: 4	Dizziness, insomnia, colic, and weight gain: 5	6 months
Schnapp et al. (2022) [15]	**Non-specified AE: 2	**Non-specified AE: 1	3 months
Aran et al. (2019) [19]	Total: 29, Sleep disturbances: 8, Restlessness: 5, Nervousness: 5, Loss of appetite: 5, Gastrointestinal symptoms: 4, Unexplained laugh: 4, Mood changes: 3, Fatigue: 3, Nocturnal enuresis: 2, Gain of appetite: 2, Weight loss: 2, Weight gain: 2, Dry mouth: 2, Tremor: 2, Sleepiness: 1, Anxiety: 1, Confusion: 1, Cough: 1, **Psychotic event: 1	NR	Mean ± SD: 10.9 ± 2.3 months
Barchel et al. (2019) [18]	Somnolence: 12, Appetite decrease: 6, Appetite increase: 4, Insomnia: 2, Sense abnormality response (to temperature): 2, Eyes blinking: 2, Diarrhea: 2, Hair loss: 1, Nausea: 1, Confusion: 1, Acne: 1, Palpitations: 1, Urinary incontinence: 1, Eye redness: 1, Constipation: 1	NR	Median (range): 66 (31-588 days)
Hacohen et al. (2022) [16]	**5 Increased aggression, **3 Anxiety, **1 Weight gain, **1 Abdominal pain, **1 Hyperactivity, **1 Decrease in communication	NR	6 months
Bar-Lev Schleider et al. (2019) [17]	Restlessness: 6, Sleepiness: 3, Psychoactive effect: 3, Increased appetite: 3, Digestion problems: 3, Dry mouth: 2, Lack of appetite: 2, **5 Stopped treatment due to non-specified AE	NR	6 months

Risk of Bias Assessment

**Figure 2:**
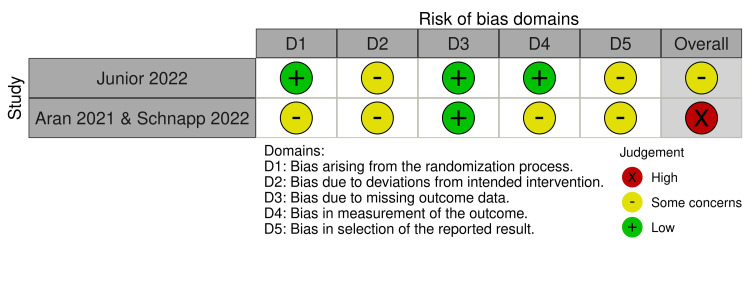
RoB-2 RoB-2: Risk of Bias-2

**Figure 3:**
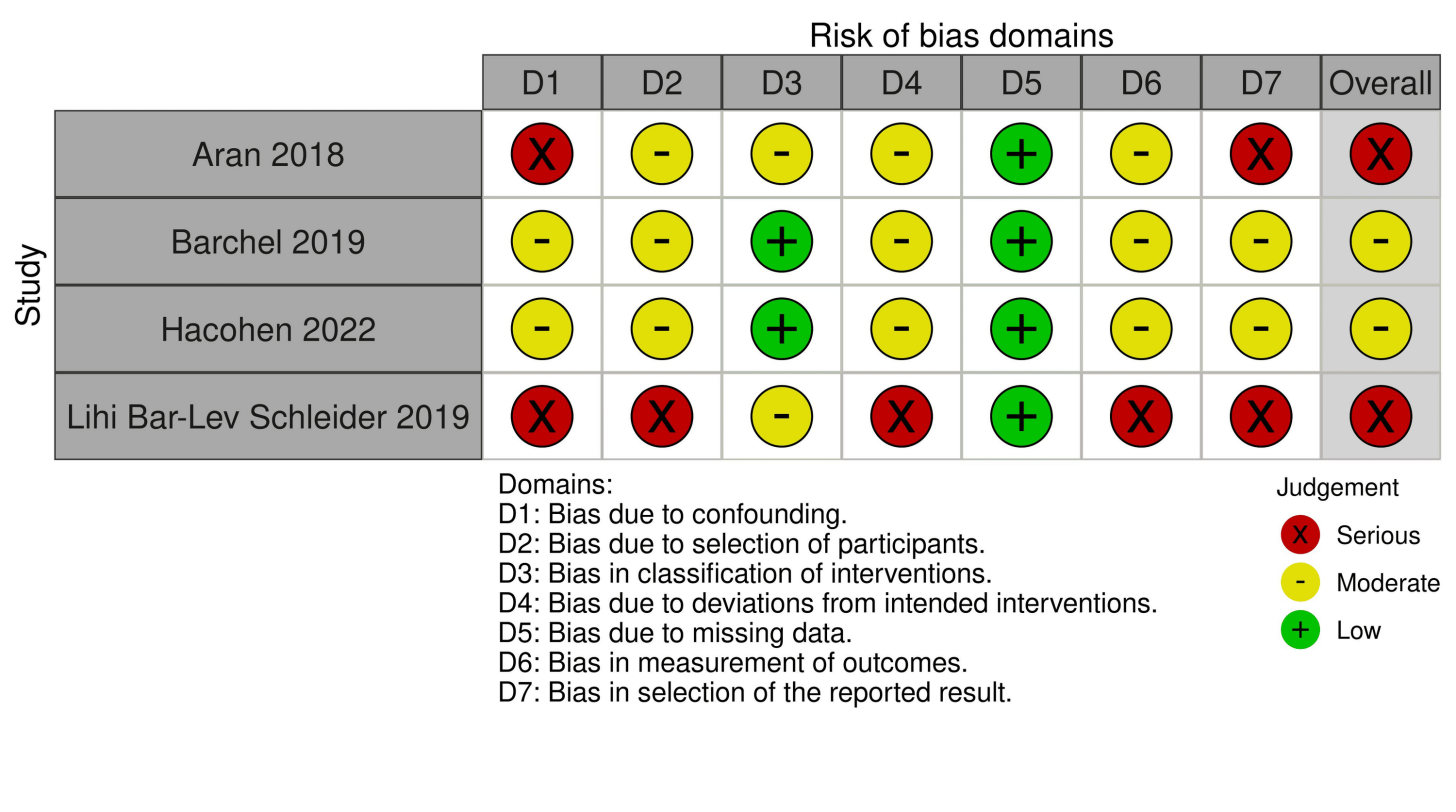
ROBINS-I ROBINS-I: Risk of Bias in Non-randomized Studies of Interventions tool

The risk of bias assessment revealed varying levels of bias across the included studies. Among the RCTs (assessed using RoB 2), Junior et al. [14] had an overall "some concerns" rating due to deviations from intended interventions and selection of reported results, while Aran et al. [3] and Schnapp et al. [15] had a "high" risk of bias, mainly due to concerns in randomization and measurement of outcomes (Figure 2). For the non-randomized studies (assessed with ROBINS-I), Barchel et al. [18] and Hacohen et al. [16] showed a moderate risk of bias, particularly in confounding and deviations from intended interventions (Figure 3). In contrast, Aran et al. [19] and Bar-Lev Schleider et al. [17] had a serious risk of bias, primarily due to confounding, selection of reported results, and measurement of outcomes, highlighting potential limitations in the reliability of their findings.

Discussion

This systematic review explored the efficacy and safety of cannabinoids, alone or compared to placebo, in managing autism symptoms in children and adolescents. Our analysis incorporated data from seven studies, including 494 patients, and focused on key clinical outcomes, such as sleep, autistic core symptoms, anxiety, behavior, social effects, and adverse events.

Preliminary evidence suggests that CBD-rich formulations may hold promise for managing certain ASD symptoms. Improvements were noted in anxiety, sleep quality, social effects, and behavior, although these findings varied across studies. Importantly, adverse events were generally mild and occurred at similar rates in cannabinoid and placebo groups, supporting the intervention’s safety profile.

Regarding sleep, studies indicate a slight improvement in sleep quality, reflected in better well-being scores (e.g., CSHQ) [16]. These findings align with recent literature emphasizing CBD’s potential role in sleep regulation [18]. Additionally, autistic core symptoms showed positive responses, with improvements in scores such as SRS-2, ATEC, and CARS, suggesting enhanced behavior and social interaction [19]. Some studies also reported reductions in hyperactivity, agitation, self-injurious behavior, and epilepsy, along with improvements in communication skills, attention, and eye contact [20-22].

Anxiety reduction was another notable finding, with results from Junior et al. [14] and Schnapp et al. [15] corroborating broader evidence of cannabinoids' anxiolytic effects. Some studies also explored the use of CBD-enriched formulations in ASD patients with ADHD, showing promising outcomes [23,24]. While a range of side effects was reported, somnolence was the most frequently observed, consistent with existing literature [24].

Research on cannabinoids' effects on core autism symptoms - such as social communication deficits and repetitive behaviors - has yielded mixed results [25]. Some studies suggest potential improvements in these areas, while others find limited impact. For instance, a systematic review highlighted that changes in core symptoms were scarcely explored, with only one study reporting some improvements in communication and social interaction in a small sample of Brazilian children with ASD [25]. While some studies reported improvements in SRS-2, ATEC, and CARS scores, suggesting enhanced social interaction and behavior, others found no significant differences when compared to placebo [16,19]. These results align with previous research indicating that cannabinoids may have a mild effect on core ASD symptoms but are not a definitive treatment [25]. The mechanisms behind these potential benefits could be linked to CBD’s interaction with the ECS, which plays a role in neurodevelopment and social behavior [25,26].

The safety profile of cannabinoid treatments in ASD populations has been generally favorable, with most adverse events reported as mild to moderate. Common side effects include drowsiness, decreased appetite, weight loss, anxiety, and restlessness. Despite these occurrences, cannabinoids are often considered well-tolerated compared to traditional pharmacological interventions. Nonetheless, careful monitoring and individualized dosing are essential to minimize potential risks, and further research is necessary to fully elucidate the long-term safety of cannabinoid use in ASD treatment [26,27].

Mechanistically, CBD interacts with the ECS, a critical signaling network involved in neurodevelopment, social behavior, and homeostasis. Emerging evidence also suggests its role in gut-brain communication, further linking it to ASD pathophysiology [25,26]. Preclinical research, such as the study by Poleg et al., demonstrated reductions in repetitive behaviors and anxiety-related symptoms in ASD mouse models following CBD treatment [8]. Additionally, low doses of THC in specific formulations appear beneficial for social behaviors, though their long-term effects remain uncertain. These findings support the potential of personalized cannabinoid therapies tailored to ASD subtypes.

Several meta-analyses have examined cannabinoids in conditions like epilepsy and multiple sclerosis, but systematic reviews and meta-analyses specifically addressing ASD remain scarce [28-30]. This study contributes to the growing body of evidence and underscores the need for further rigorous research.

This study presents several limitations. First, the heterogeneity of the included studies - particularly regarding intervention types, dosing regimens, and outcome measures - complicates direct comparisons and meta-analysis. The impossibility of carrying out a meta-analysis is due to the lack of essential statistical data in the primary studies, such as means, standard deviations, and interquartile ranges. Second, the small sample sizes and substantial missing data further limit the reliability of the findings. Third, variation in cannabinoid formulations across studies makes it difficult to assess their true efficacy and safety. Additionally, methodological biases, particularly in patient selection and randomization, may have influenced results. Finally, the limited number of high-quality RCTs on this topic restricts the strength of the conclusions drawn.

Future research should focus on standardized methodologies, larger and more diverse patient populations, and long-term safety evaluations. Further studies should also explore optimal dosing regimens and the biological mechanisms underlying cannabinoids' effects in ASD.

## Conclusions

This systematic review assessed the potential impact of cannabinoids on ASD symptoms and adverse effects. While preliminary evidence suggests potential benefits, particularly for anxiety, sleep, and behavior, the findings remain inconclusive due to study heterogeneity and methodological limitations. More rigorous, well-designed RCTs are necessary to confirm these results and establish clear treatment guidelines for cannabinoid use in ASD.
